# Experimental and Modeling Study of Ethylene Glycol–Water Mixture Dehydration by Pervaporation for Coolant Liquid Recovery

**DOI:** 10.1021/acsomega.5c12501

**Published:** 2026-04-14

**Authors:** Daniel Gorri, Alvaro Martín-Quijano, Mireya Revuelta, Iñigo Baro, Raúl González-Martín, Daniel González-Revuelta, Marcos Fallanza, Alfredo Ortiz

**Affiliations:** 1 Departamento de Ingenierías Química y Biomolecular, 16761 Universidad de Cantabria, Av. de los Castros s/n, 39005 Santander, Spain

## Abstract

The dehydration of ethylene glycol–water mixtures is a relevant challenge in the recovery and reuse of cooling liquids from industrial processes, particularly in the pharmaceutical sector. This work investigates the feasibility of using pervaporation with a hydrophilic polymeric membrane (PERVAP 4101, based on highly cross-linked poly­(vinyl alcohol)) to partially dehydrate mixtures containing up to 70 wt% water and maintain compositions close to the eutectic point (≈58 wt% ethylene glycol). Experimental tests were conducted in a laboratory-scale pervaporation unit at 60, 70, and 80 °C. The influence of feed composition and temperature on permeation flux, separation factor, and long-term stability was analyzed. A conditioning stage at 80 °C was found to be essential to achieve steady operation, resulting in high water selectivity (98.9–99.8 wt% water in permeate) and stable fluxes up to 4.2 kg m^–2^ h^–1^ during 30 h continuous tests. ATR–FTIR spectra of the pristine and conditioned membranes revealed only minor structural modifications, suggesting a slight reduction in crystallinity associated with increased permeability, which was later confirmed by DSC analysis. The experimental data were successfully correlated using a solution–diffusion model, considering the dependence of permeance on component activity and temperature. The apparent activation energies for water and ethylene glycol permeation were 34–42 and 43–58 kJ mol^–1^, respectively. Results confirm that PERVAP 4101 membranes provide an efficient and stable route for ethylene glycol dehydration at high water contents, demonstrating potential for industrial implementation.

## Graphical abstract

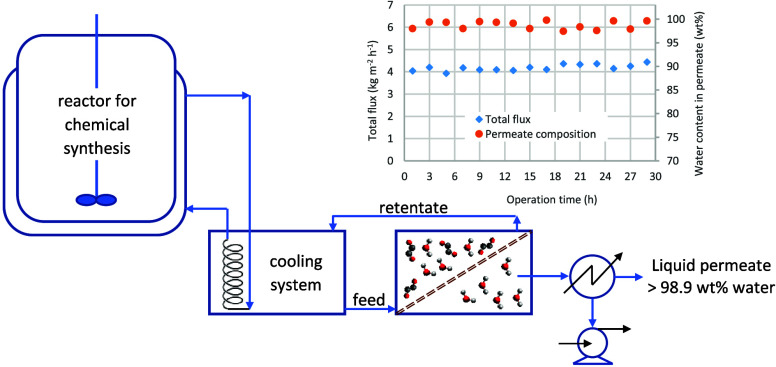

## 1 Introduction

Organic solvents are essential in the chemical and pharmaceutical industries, facilitating reactions, extractions, and purifications. They enable the synthesis of complex compounds, ensuring high efficiency and product quality. However, their use demands careful handling due to environmental and health concerns, highlighting the importance of sustainable practices like solvent recycling and recovery.^1^ Additionally, there are other organic compounds used in utilities that may eventually generate waste streams. This is the case of ethylene glycol, whose aqueous mixtures are employed in industrial cooling systems, which is the subject of focus in this study.

Ethylene glycol–water mixtures are widely used in cooling systems due to their excellent heat transfer and antifreeze properties. These solutions prevent freezing and overheating in equipment, ensuring efficient operation across temperature extremes. A key characteristic is the eutectic point, occurring at approximately 58 wt% ethylene glycol, where the freezing temperature reaches its minimum of around −50 °C.^2^ This feature ensures reliable operation in cold environments, preventing freezing and protecting equipment from thermal stress.

In many chemical synthesis processes taking place in the pharma industry, which are characterized by performing a sequence of reaction steps in batch systems, they may require heating with steam through the reactor jacket in one stage of the process, and subsequently another cooling stage using aqueous glycol mixtures as coolant liquids. Due to the manipulation of these streams during the operation of batch processes, the water content in glycol mixtures can increase to undesirable levels (≈70%), thus moving away from the eutectic point. Therefore, it is interesting to have a versatile tool to adjust the water content of this type of mixtures. The separation of EG/water mixtures is also a necessary operation in the glycolysis process for regenerating waste PET and polyester manufacturing, as well as in the production of EG from the hydrolysis of ethylene oxide.^3,4^

Several unit operations are available for the dehydration of aqueous mixtures of organic solvents, each with distinct advantages and limitations. Distillation is the most established method, relying on boiling point differences to separate water. It is effective and widely scalable but becomes energy-intensive when dealing with azeotropic mixtures or heat-sensitive compounds. In such cases, specialized techniques like azeotropic or extractive distillation are required, often involving the addition of entrainers, which can complicate the process and increase costs.^5,6^ Adsorption is another option, utilizing materials such as alumina or zeolites to selectively remove water.^7–9^ This method is effective for low-volume streams and can achieve high levels of dryness, but the need for adsorbent regeneration increases operational complexity and costs. The third option, membrane pervaporation, stands out for its modular nature and operational flexibility. It involves the selective permeation of water through a dense membrane, followed by its evaporation on the permeate side which is maintained under vacuum.^10–12^ Unlike distillation, pervaporation does not require entrainers for azeotropic mixtures, simplifying the process. Additionally, its modular design allows for easy scaling and adaptation to varying process conditions, making it an attractive option for diverse industrial applications. The choice between these methods depends on the process requirements, economic factors, and specific properties of the mixture to be dehydrated.

Among the possible applications of pervaporation for the separation of liquid mixtures, solvent dehydration is the application that has reached the highest technology readiness level (TRL), having been reported for the dewatering of numerous solvents, as documented in several review papers.^13–16^ More specifically, several studies have been published in the last decades on the separation of ethylene glycol/water mixtures, comprehensively summarized in the review paper by Rostovtseva et al.^17^ A literature analysis shows that most reported studies correspond to the separation of mixtures with a water content of up to 20 wt% and among the polymers used to prepare the selective membranes, poly­(vinyl alcohol) (PVA) and, to a lesser extent, chitosan clearly stand out.

This work explores the use of pervaporation with hydrophilic polymeric membranes to partially dehydrate aqueous mixtures of ethylene glycol and maintain their composition close to the eutectic point. In the proposed case study we face a double challenge, which leads to establishing whether the membrane is stable at such high water contents (up to 70% water content), and what is the highest operating temperature that allows stable operation of the membrane. For this reason, a hydrophilic PVA-based membrane with a high degree of cross-linking was selected, and a series of experiments were performed to evaluate the influence of operating temperature and feed composition on the separation performance, also checking the long-term behavior of the membrane.

## 2 Results and Discussion

### 2.1 Membrane Conditioning and Separation Feasibility

In the first stage, the study focused on evaluating the viability of the pervaporation process for processing liquid mixtures with a water content of around 70 wt%. Many hydrophilic polymer membranes are not stable in contact with fluid phases with a high water content, and this problem tends to worsen as the operating temperature increases. For this reason, a series of experiments were initially carried out (in duplicate) following a sequence of increasing temperatures: 60 °C, 70 °C, and 80 °C. As expected, the permeation flux increased significantly with operating temperature. When the sequence of experiments was repeated (once again) with the same membrane, it was observed that the fluxes at 60 and 70 °C had increased appreciably compared to the initial experiments, while maintaining a high selectivity toward water. This indicated that the membrane required a conditioning stage at 80 °C to subsequently achieve its best performance. To confirm this finding, experiments were repeated with another membrane sample, before and after being stabilized at 80 °C. As can be seen in Figure 1, conditioning the membrane with a feed liquid mixture at 80 °C allows the membrane to be stabilized to obtain high permeate fluxes with a high separation factor, with a performance that is maintained over time, as will be shown later with long-time experiments. Since the experiments were conducted in batch mode, water permeation through the pervaporation membrane causes the water concentration in the recirculation tank to decrease over time. The reported feed composition corresponds to the average of the feed compositions measured at the beginning and at the end of the permeate sampling interval (typically 30 min). The most notable change in membrane behavior occurred with the experiments carried out at 60 °C where, as shown in Figure 1a,b, with the new membranes (without conditioning) permeation fluxes in the range of 0.52–0.77 kg m^–2^ h^–1^ and water contents in the permeate of 98.5–100 wt% were obtained. After carrying out the conditioning at 80 °C for at least 8 h, when working again at 60 °C permeation fluxes in the range of 1.5–2.1 kg m^–2^ h^–1^ were obtained, while the water content in the permeate remained at 98.9–99.8 wt%. The fact that permeate flux values were obtained in a relatively wide range is due to the fact that the experiments were carried out in batch mode and therefore the composition of the feed mixture varies throughout each experiment. For this reason, each experimental permeation flux value must be interpreted in relation to its corresponding feed mixture composition, as shown in Figure 1. The difference between the results obtained at 70 °C with membranes before and after conditioning is also significant, with 65–80% higher fluxes obtained while the water content in the permeate remained above 98.5 wt% in most permeate samples (Figure 1c,d).

**Figure 1:**
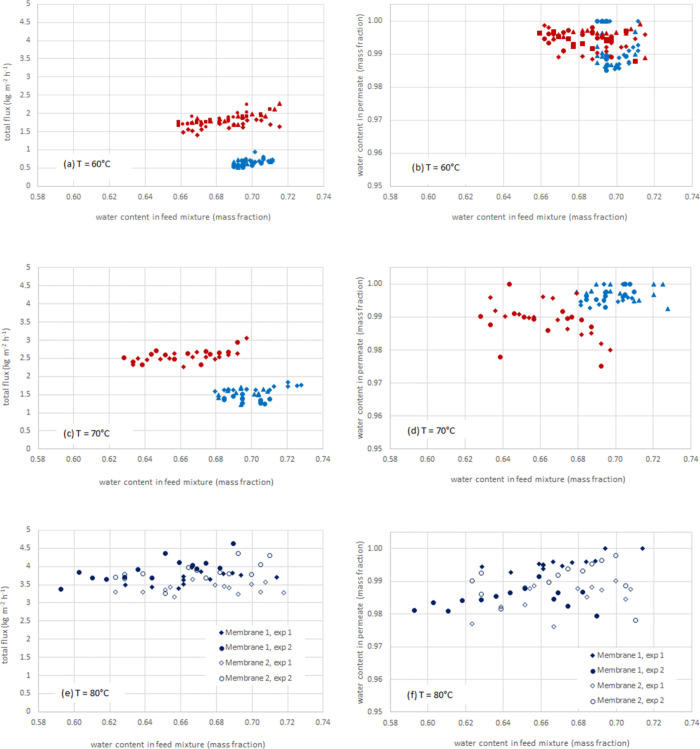
Permeation fluxes (a, c, e) and permeate composition (b, d, f) at three operating temperatures (60, 70, and 80 °C), obtained with the membrane before (blue symbols) and after conditioning at 80 °C (red symbols).

In order to assess potential changes in separation performance over the operating time, a long-term test with the preconditioned membrane was conducted under continuous operation for 30 h using a feed mixture with a high water content. The test was carried out at 80 °C, and to maintain the operating conditions as stable as possible, the collected permeate was recycled back to the feed tank (noting that only a minimal sample volume was withdrawn for refractometric analysis). Thus, the feed mixture composition was maintained at 67.0 ± 1.6 wt% water throughout the test. Figure 2 presents the separation performance indicators over the course of the operation time. The water concentration in the permeate (98.7 ± 0.84 wt% water) and the total flux (4.18 ± 0.14 kg m^–2^ h^–1^) remained nearly constant throughout the entire testing period, indicating the membrane’s long-term stability.

**Figure 2:**
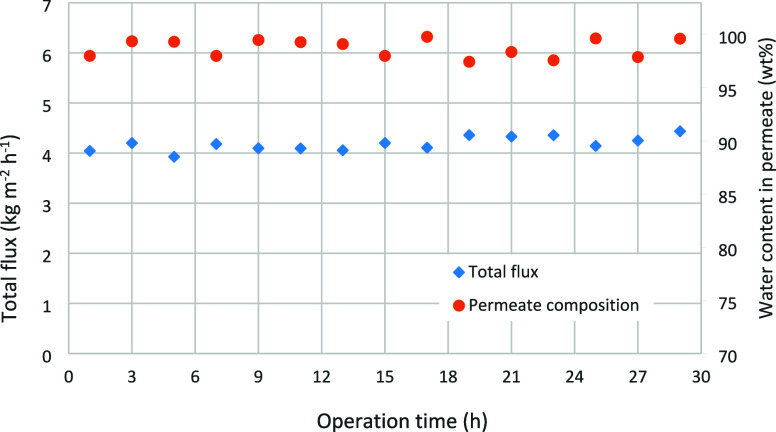
Long-term permeation test at 80 °C with the previously conditioned membrane.

The observed experimental behavior raises questions regarding the changes that occurring in the membrane structure during conditioning. Several studies reporting information on the conditioning of pervaporation membranes have been published since the pioneering works of Ping et al.^18^ and Marin et al.^19^ However, it cannot be stated that there is a unique pattern of behavior since it depends on the membrane material, the composition of the liquid mixture, and the operating temperature. An initial transient stage with a decrease in permeation flux and an increase in selectivity is frequently observed due to a simple compaction phenomenon of the polymer chains that make up the membrane.

Among the few published works that studied the conditioning of PV membranes for the separation of ethylene glycol/water mixtures, the work by Feng and Huang^20^ is worth mentioning. These authors studied the behavior of a chitosan membrane synthesized in their laboratory, in contact with a feed mixture containing 10 wt% water. Pervaporation experiments were initially carried-out by systematically increasing the operating temperature from 22 to 80 °C, and then the membrane was held at 80 °C for five days to be “conditioned” before another series of measurements in which the temperature was systematically decreased. As a result of the conditioning process, experimental results showed a moderate decline in permeation flux while improving water selectivity (as water content in permeate). The authors reported no visible changes in the membrane cross-section based on SEM analysis, suggesting that the morphological changes were likely due to subtle rearrangements within the polymer matrix.

Figure 3 presents cross-sectional SEM images of the membranes used in this study: (a) a new, unconditioned membrane and (b) a membrane after conditioning at 80 °C, which was subsequently employed in the permeation experiments. In both cases, a thin, dense PVA-based selective layer is deposited onto a porous PAN support. The only noticeable difference between the two membranes is a slight compaction of the dense top layer in the conditioned membrane. Based on the SEM images, the thickness of the top layer was estimated to be 2.6 μm for the new membrane and 1.8 μm for the conditioned membrane. In addition, surface SEM images revealed that both membranes exhibit smooth, defect-free surfaces.

**Figure 3:**
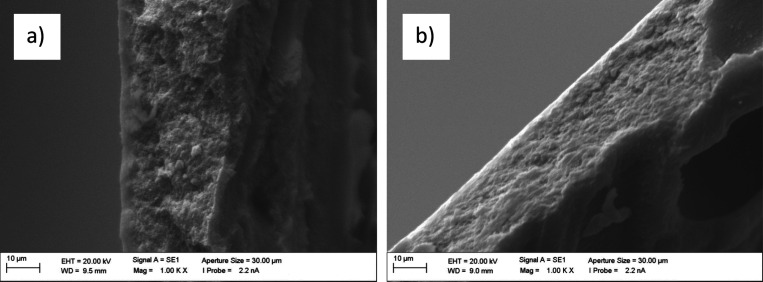
SEM images of the cross-section of (a) new, unconditioned membrane; (b) membrane after conditioning at 80 °C.

Poly­(vinyl alcohol) is a semicrystalline polymer with a glass transition temperature (*T*_*g*_) of 85 °C.^21^ Various studies in the literature suggest that in PVA-based membranes, transient phenomena induced by temperature and/or solvent exposure may occur, leading to changes in the crystallization of the polymer.^18,22^ As the membrane swells, larger segments of the polymer chains gain increased mobility, which can facilitate the reorganization of the chains into a more stable crystalline structure. Crystallinity significantly affects the dissolution of the feed mixture in the membrane during pervaporation, as this process (like diffusion) occurs exclusively in the amorphous regions of the polymer. In order to evaluate possible changes in the membrane, FTIR spectra of the pristine membrane and the membrane after conditioning at 80 °C were recorded, as shown in Figure 4. It is known that the percentage of crystallinity of uncrosslinked PVA films is proportional to the intensity of the peak at 1142 cm^–1^,^23,24^ although it has also been reported that the position and/or intensity of the peaks can change if the polymer is subjected to cross-linking.^25^ From Figure 4, it can be observed that the membrane spectra before and after conditioning are quite similar, with no apparent peak shift or new peaks. Additionally, these spectra are in agreement with those reported by Esmaeili and Kirk^26^ for the Pervap 4101 membrane. The ratio of the absorbances at 1142 cm^–1^ and 1416 cm^–1^ suggests a slight reduction in the crystallinity of the polymer in the conditioned membrane relative to the pristine, unconditioned membrane. This reduction in crystallinity is associated with an increase in membrane permeability.

**Figure 4:**
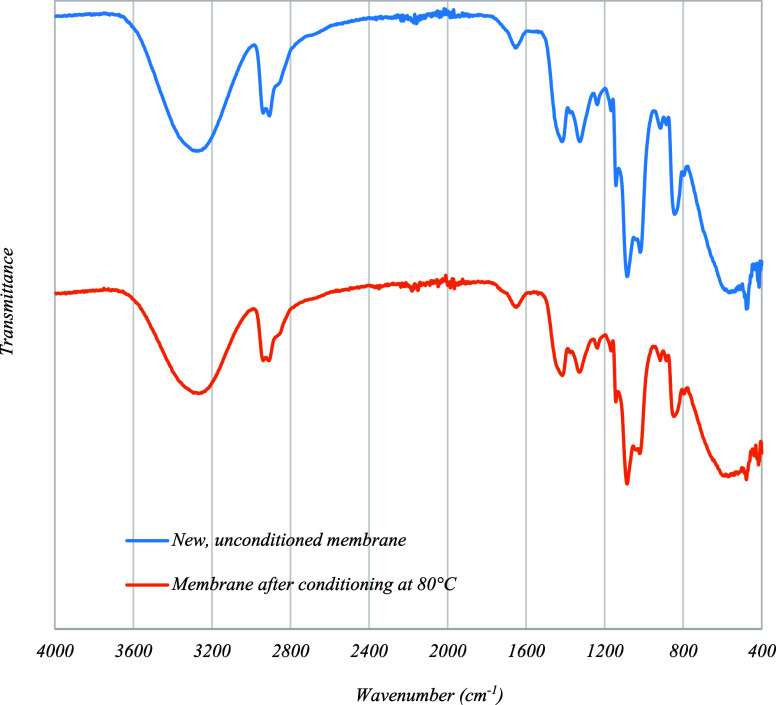
FTIR spectra of the top selective layer of the membranes.

To confirm the structural changes in the membrane, thermal analyses were carried out using differential scanning calorimetry (DSC). For this purpose, the dense top-layer was carefully detached and separated from both the pristine membrane and the membrane conditioned at 80 °C and subsequently used in the permeation experiments. Figure 5 presents the DSC thermograms of both samples. At temperatures below 100 °C, both samples exhibit an initial endothermic peak, which is likely associated with moisture loss. On the other hand, an additional endothermic peak with a maximum at 184.5 °C is observed exclusively in the conditioned membrane and is attributed to the evaporation of residual ethylene glycol. Finally, both samples display an endothermic peak at approximately 202 °C, corresponding to the melting transition of the crystalline domains of PVA.^24^ To further investigate the membrane crystallinity, and following the methodology reported in the literature,^24,27^ the melting enthalpies were determined from the DSC thermograms, and the degree of crystallinity of the membranes was calculated using eq 1:

$$X_{c}=\frac{\Delta H_{f}}{\Delta H_{f}^{0}}\times 100$$ 1

where *X*_*c*_ is the degree of crystallinity (%), Δ*H*_*f*_ is the enthalpy of fusion from the endothermic melting peak (J g^–1^) and Δ*H* ^*0*^_*f*_ is the enthalpy of fusion of the totally crystalline polymer (138.6 J g^–1^).^28^ Accordingly, the enthalpies of fusion were determined from the DSC thermograms, yielding values of 46.6 J·g^–1^ for the new membrane and 25.2 J·g^–1^ for the conditioned membrane. These values correspond to degrees of crystallinity of 33.6% and 18.2%, respectively. Therefore, these results are consistent with the FTIR analysis and further support the hypothesis that the observed increase in membrane permeability is associated with a reduction in polymer crystallinity within the dense selective layer.

**Figure 5:**
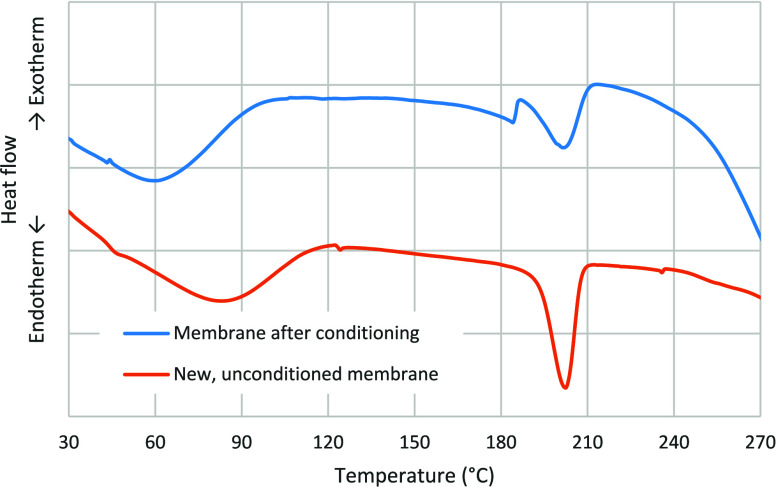
DSC thermograms of the top dense layer of the membranes.

### 2.2 Influence of the Operating Conditions on the Membrane Separation Performance

Although the primary objective of this study is to assess the feasibility of using pervaporation to dehydrate ethylene glycol/water mixtures in order to maintain their composition near the eutectic point, it was also considered important to extend the investigation to a broader range of concentrations, which could be relevant for other applications. Accordingly, a series of experiments were performed using a membrane previously conditioned at 80 °C with mixtures containing approximately 70 wt% water. The tests were conducted at three temperatures (60, 70, and 80 °C) with water/ethylene glycol mixtures of increasing water content, ranging from 5 wt% to 70 wt%. It is well-known that the separation performance of pervaporation depends not only on the membrane characteristics but also on operating parameters such as feed composition, temperature, and other factors. Since the sorption and diffusion of each component are concentration-dependent, variations in feed composition influence both flux and selectivity. Furthermore, changes in operating temperature may alter the membrane structure and the mutual interactions between components, thereby affecting their mass transport coefficients.^29^

In the pervaporation of binary liquid mixtures, a nonlinear relationship typically exists between the permeation flux and the component concentration in the feed, due to the complex interactions between the membrane material and the permeating species.^30,31^ Figure 6 illustrates the variation of the water permeation flux obtained from the dehydration experiments as a function of the water content in the feed. Each data point shown in the plot represents the average value of several measurements performed under the same operating conditions. For all tested temperatures, the permeation flux increases with increasing water content in the feed. For instance, at 80 °C, as the water concentration rises from 20 to 40 wt%, the total flux and water flux increase from 1.02 to 2.43 kg·m^–2^ h^–1^ and from 0.89 to 2.31 kg·m^–2^ h^–1^, respectively. The permeation flux of water is markedly higher than that of ethylene glycol. Due to the presence of hydroxyl (-OH) groups, PVA exhibits strong hydrophilicity. Although both water and ethylene glycol are polar molecules, the polymer shows a greater affinity for water, as evidenced by swelling tests reported in the literature.^3,32^ Consequently, as the water content in the feed mixture increases, the polymer matrix becomes more swollen, inducing a plasticization effect on the membrane. This phenomenon enhances the diffusion of permeating species through the membrane.

**Figure 6:**
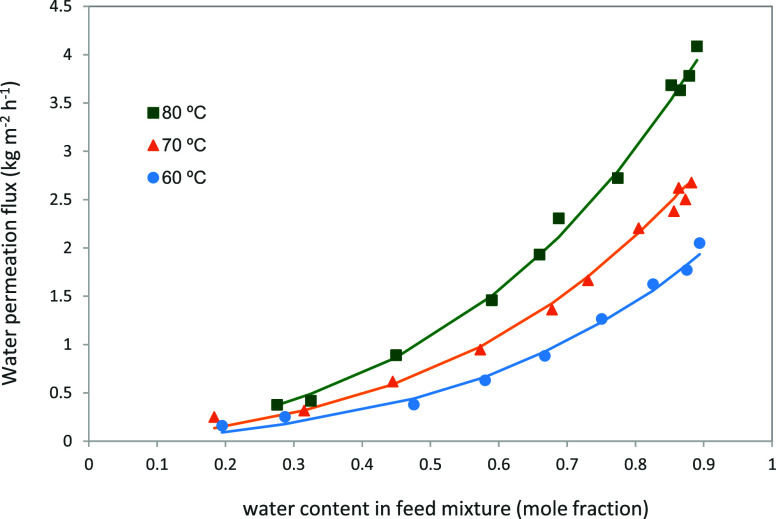
Water flux vs water content in the feed mixture. The lines represent the simulated fluxes at each temperature.

The separation factor (α) is also influenced by the feed composition. However, in contrast to the permeation flux, the separation factor only increases moderately with increasing water concentration in the feed. Thus, for the experiments at 80 °C mentioned above, when the feedwater concentration increases from 20 to 40 wt% the separation factor changes from 29.4 to 32.7, reaching a value of 43 for feed mixtures with 70.2 wt% water. The increased swelling of the membrane upon contact with water facilitates the permeation of both components, resulting in a slight improvement in the separation factor.

For a feed mixture containing 20 wt% water, the water flux through the membrane reached 0.38, 0.62, and 0.89 kg·m^–2^ h^–1^ at operating temperatures of 60, 70, and 80 °C, respectively. A progressive increase in the overall permeation flux was observed with rising temperature. This behavior can be attributed to two main factors. First, higher temperatures increase the vapor pressure of the components, thereby enhancing the driving force for permeation. Second, the increase in segmental mobility of the polymer chains with rising temperature enhances the diffusion of permeating species. Consequently, the transport of both permeants is promoted, resulting in an overall increase in the total permeation rate.

Empirically, the temperature dependence of the permeation flux follows an exponential trend, as illustrated by the semilogarithmic plot in Figure 7. The water flux at three different temperatures is plotted in logarithmic form as a function of the reciprocal temperature for six different feed compositions. Qualitatively, the plots indicate that the permeation flux increases with higher water concentrations in the feed and decreases as the temperature is lowered. A similar trend was observed for the temperature dependence of the ethylene glycol flux. To describe this behavior, an Arrhenius-type equation (eq 10) was employed to represent the temperature dependence of the partial fluxes.

**Figure 7:**
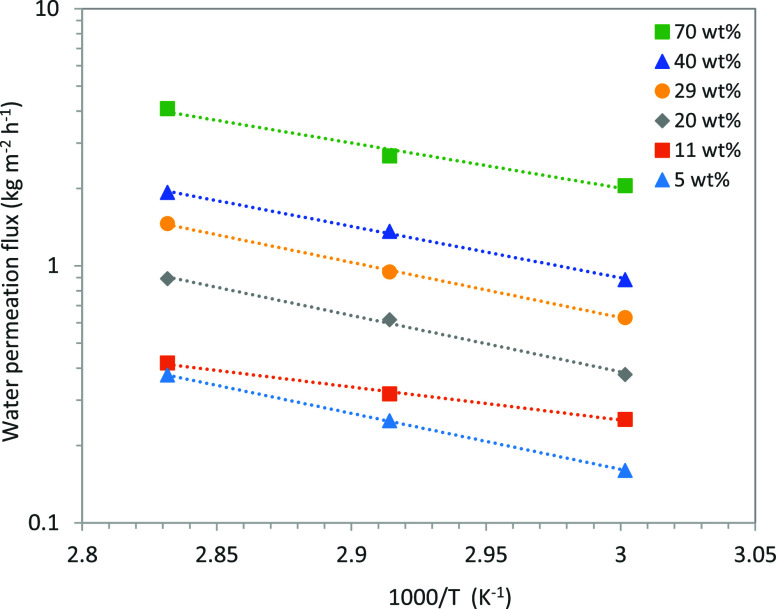
Influence of operating temperature on water flux for various feed mixture compositions.

The apparent activation energies (*E*_*J,i*_) for water and ethylene glycol permeation were estimated from experimental data obtained at different temperatures. As shown in Figure 7, the effect of temperature on the water permeation rate is nearly the same for all feed compositions. The apparent activation energy for water permeation ranges from 34 to 42 kJ·mol^–1^, while that for ethylene glycol permeation ranges from 43 to 58 kJ·mol^–1^.

Among the studies on water/ethylene glycol separation that examine membrane performance when working with mixtures with high water content, those conducted by Feng and Huang^20^ (using chitosan membranes), Chen and Chen,^33^ and Guo et al.^3^ are particularly noteworthy, the latter two employing cross-linked PVA membranes. Recent studies have reported interesting results using advanced materials, such as those using new polymeric materials,^35^ incorporating graphene oxide into the composite membrane,^36^ or using hydrophilic derivatives from the polymer of intrinsic microporosity (PIM-1).^4^ Table 1 presents a comparison of results obtained for feed mixtures with high water content. It is evident that ensuring a sufficiently thin selective dense layer is crucial for achieving meaningful permeate fluxes. It can be observed that the Pervap 4101 membrane is among those that exhibit the highest permeate fluxes while maintaining a high water content in the permeate. These studies are consistent in showing that the separation factor decreases with increasing operating temperature. This behavior is governed by the relative values of the activation energies *E*_*j,water*_ and *E*_*j,EG*_, which respectively describe the temperature-dependence of individual permeation fluxes *J*_*water*_ and *J*_*EG*_.

**Table 1:** Comparative Performance of PV Membranes for Ethylene Glycol Dehydration from Mixtures with High Water Content

membrane	thickness (μm)^a^	feed water concentration (wt%)	operating temperature (°C)	flux (kg m^–2^ h^–1^)	water content in permeate (wt%)	separation factor	reference
chitosan/polysulfone composite membrane	N/A	70	35	3.2	99.5	85	^20^
cross-linked PVA	N/A	69.6	60	0.47	99.5	88	^33^
		69.5	70	0.63	99.4	72	
		69.4	80	1.17	99.4	73	
cross-linked PVA	15 ± 3	60	70	0.87	99.97	2280	^3^
		80	70	1.29	99.995	5509	
Hyflon	86 ± 3	70.6	35	0.63	95.4	8.7	^34^
		70.4	50	0.96	94.6	7.3	
		70.6	65	1.44	93.9	6.4	
PDMAEMA/PSF composite membrane	1	66.8	30	5.43	98.5	32.6	^35^
PPO/GO (0.7%) composite membrane	3 ± 0.2	70	22	0.35	96.7	13	^36^
AO-PIM	<10	70	25	3.0^b^	99.3	62	^4^
		70	45	6.3^b^	98.5	28	^4^
		70	65	9.1^b^	99.0	43	^4^
DAT-PIM	<10	70	25	1.4^b^	99.4	69	^4^
		70	45	2.6^b^	99.1	46	^4^
		70	65	4.9^b^	99.4	68	^4^
Pervap 4101	2.6	71	60	2.06	99.5	88	this work
		70	70	2.95	98.9	39	
		70.2	80	4.13	99.0	43	

a Thickness of the dense selective layer.

b Thickness-normalized total flux (μm kg m^–2^ s^–1^).

The enrichment factor (β) is defined as the ratio of concentrations of the preferentially pervaporating species in permeate and feed (eq 4). For process design, some authors propose to use this parameter in the formulation of the mathematical equations governing the separation performance.^21^ Figure 8 shows the experimental enrichment factor as a function of the feedwater concentration for the Pervap 4101 membrane. It is observed that the enrichment factor is almost independent of the temperature, which simplifies its use in design calculations.

**Figure 8:**
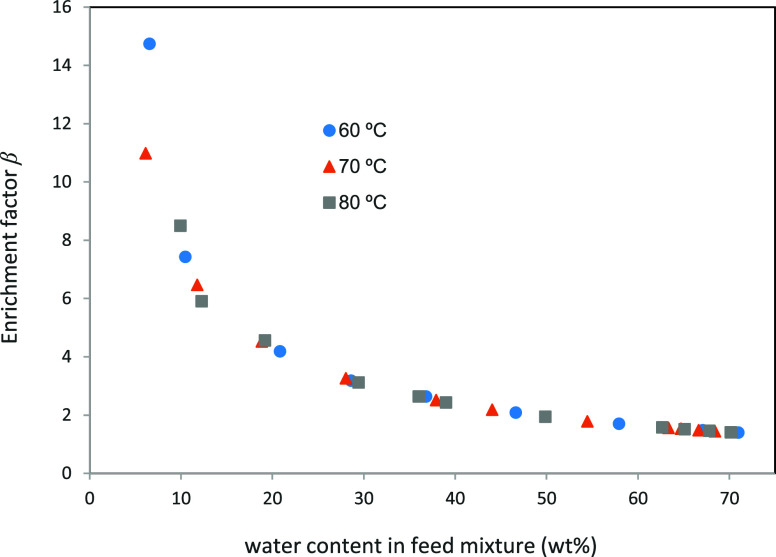
Enrichment factor as a function of the feedwater concentration.

The flux data of the individual components, water and ethylene glycol, were fitted to the mathematical model described in Section 4.4. The experimental data were correlated using eqs 5–8 to determine the values of the characteristic mass transfer parameters and to evaluate the validity of the proposed model. The best-fit parameter values, obtained using 60 °C as the reference temperature, are summarized in Table 2. Figure 6 compares the experimental values of the water fluxes with the corresponding simulated results at different temperatures. The experimental results show good agreement with the model predictions, thereby supporting the main assumptions of the model. The permeance of both water and ethylene glycol in all mixtures decreases with increasing temperature, which is a common behavior observed in pervaporation of aqueous systems.^34^ Consequently, the activation energies associated with the permeance of both permeating species are negative in all cases.

**Table 2:** Parameters of the PV Model for Water/Ethylene Glycol Mixtures Permeating through PERVAP 4101 Membranes (60 °C)

	water	ethylene glycol
*A*_*i*_	130.92	1851.2
*B*_*i*_	1.8115	0.0639
*E*_*Q,i*_ *(kJ mol^–1^)*	–7.6	–36.9

The results obtained in this study confirm the technical feasibility of using pervaporation to dehydrate coolant liquids with high water content. It is known that other conventional separation techniques, such as distillation or adsorption, could be used for the same purpose. The advantages and disadvantages of each of these techniques have already been presented in the Introduction section, in general terms with respect to the dehydration of organic solvents. With specific regard to the dehydration of EG/water mixtures with high water content, it should be noted that a mixture containing 70 wt% water boils at 103.3 °C under atmospheric pressure, and the boiling point increases with the glycol concentration, reaching 109.6 °C at the eutectic point (42 wt% water). By contrast, dehydration can be efficiently achieved by pervaporation at moderate temperatures (60–80 °C). Consequently, pervaporation requires a smaller amount of the auxiliary heating fluid (typically steam), whereas, due to the necessary reflux during the distillation process, energy consumption will be significantly higher.^37^ Furthermore, as reported by Shah and Bartels^38^ in their study on EG/water separation, membrane-based processes do not scale up as economically as distillation because of their inherently modular nature. Hence, the use of pervaporation is constrained by process scale. On the other hand, the potential use of adsorption is limited, as frequent regeneration cycles would be required due to the high water content of the streams to be treated. By contrast, pervaporation stands out for its versatility and can be operated periodically or on a demand-driven basis, since water contamination in coolant liquids is difficult to predict.

## 3 Conclusions

This study demonstrates the feasibility and robustness of pervaporation using a highly cross-linked poly­(vinyl alcohol) membrane (PERVAP 4101) for the dehydration of ethylene glycol–water mixtures containing up to 70 wt% water. The process allows the removal of excess water to keep the mixture close to the eutectic composition, which is essential for the reuse of glycol-based coolants in industrial and pharmaceutical applications. Experimental results confirmed that operating temperature and feed composition strongly affect the transport properties through the membrane.

A short conditioning step at 80 °C proved to be a key requirement to achieve stable operation, leading to a significant enhancement in flux while maintaining high selectivity. Once conditioned, the membrane exhibited excellent water selectivity (>98.9 wt% water in permeate) and high total fluxes reaching 4.2 kg·m^–2^ h^–1^ at 80 °C. Long-term pervaporation tests conducted for 30 h under constant conditions verified the membrane’s chemical and mechanical stability, with negligible performance decline. ATR-FTIR analysis indicated that only minor structural modifications occurred after operation, consistent with a slight reduction in polymer crystallinity and increased permeability. The hypothesis about the decrease in crystallinity was supported by DSC analysis on the dense top-layer.

The experimental data were successfully described using a global solution-diffusion model incorporating the dependence of permeance on component activity and temperature. The apparent activation energies were estimated between 34 and 42 kJ·mol^–1^ for water and 43–58 kJ·mol^–1^ for ethylene glycol, indicating a stronger temperature dependence for the latter. The agreement between model and experimental data validates the proposed approach for process prediction and design.

Overall, pervaporation with PERVAP 4101 membranes provides an efficient, energy-saving, and environmentally favorable alternative for the dehydration and recycling of ethylene glycol–water mixtures, offering strong potential for industrial implementation.

## 4 Experimental Section

### 4.1 Materials

The aqueous feed solutions for the PV experiments were prepared by mixing ethylene glycol (Labkem, analytical grade) with ultrapure water Milli Q obtained from a Merck-Millipore system (supplied by Merck KGaA, Darmstadt, Germany). A survey of commercially available membranes was made, and the PERVAP 4101 model, manufactured by DeltaMem AG (Switzerland), was selected. This membrane has a selective layer based on poly­(vinyl alcohol) (PVA) with a high degree of cross-linking, which is coated on a porous support from poly­(acrylonitrile) (PAN). This support has an asymmetric pore structure, which is in turn on a nonwoven fabric support that supplies the mechanical strength and manageability of the membrane.^39^ The high degree of cross-linking of the selective dense layer allows working with feed mixtures with water content greater than 50 wt%, provided that the working temperature does not exceed 80 °C.

### 4.2 PV Experiments

The permeation experiments were carried out using ethylene glycol/water solutions, with a water content of up to 70 wt%. The pervaporation experiments were carried out in a laboratory-scale unit, previously used by the authors in different pervaporation studies. The experimental setup is described in detail elsewhere.^30,40^ Flat membranes were inserted in a circular plate and frame pervaporation test cell, providing 0.0178 m^2^ of membrane area. The PV tests were performed at three operating temperatures (60 °C, 70 °C, and 80 °C), while the permeate zone was kept under vacuum using a diaphragm vacuum pump (Vacuubrand PC 3004 VARIO). Feed flow was maintained at a relatively high rate of about 2 L min^–1^. The tank mixture was thermostatically controlled by a heating fluid supplied from a thermostatic bath (Grant, model LT ecocool 150). Temperature was monitored both at the inlet and the outlet of the pervaporation module. The pervaporation system was provided with a sampling valve on the feed side, allowing the collection of a few milliliters of liquid samples over time. The permeate was collected at regular intervals, weighted and analyzed. The composition of water-ethylene glycol mixtures was determined using refractive index measurements at 25 °C (Maselli LR-02 Digital Refractometer).

The separation performance of the membrane was evaluated in terms of permeate flux *J*, separation factor α and enrichment factor β. The total flux *J* (kg m^–2^ h^–1^) across the membrane is obtained relating the mass of permeate collected with the time interval and the membrane area, as follows:

$$J=\frac{m}{A_{m}\Delta t}$$ 2

where *m* is the permeate mass collected (kg), *A*_*m*_ is the membrane area (m^2^) and Δ*t* is the time interval for sample collection (h).

After that, the flux for each component *J*_*i*_ is calculated from the total flux and the permeate composition obtained by refractive index measurements. Separation factor α and enrichment factor β are calculated using eqs 3 and 4, respectively:

$$\alpha_{i,j}=\frac{y_{i}/y_{j}}{x_{i}/x_{j}}$$ 3

$$\beta =\frac{\mathrm{Water}\;\mathrm{content}\;\mathrm{in}\;\mathrm{permeate}\;(\mathrm{wt}\%)}{\mathrm{Water}\;\mathrm{content}\;\mathrm{in}\;\mathrm{feed}\;\mathrm{solution}\;(\mathrm{wt}\%)}$$ 4

### 4.3 Membrane Characterization

The thickness of the dense layers and cross-sectional morphologies of the composite membranes were determined by means of scanning electron microscopy (SEM, model Zeiss EVO MA15). The samples were prepared by immersing and fracturing the membranes in liquid nitrogen, followed by gold thin film deposition using a sputter coater.

Differential scanning calorimetry (DSC) measurements were carried out using a Mettler Toledo DSC-822e system, operating under nitrogen flow of 50 mL min^–1^. For the calorimetric analysis, a heating ramp was programmed from room temperature to 300 °C at a heating rate of 10 K min^–1^. Data were collected with Mettler Toledo STAR^e^ software and analyzed in order to assess physical state transformations during processing.

Furthermore, ATR-FTIR analysis of two membrane samples, one of them corresponding to a pristine membrane and another sample taken from a membrane used in PV experiments, were carried out using a Perkin-Elmer spectrum 65 Fourier Transform Infrared Spectrometer in the region 400–3900 cm^–1^. ATR-FTIR analyses were performed to evaluate possible changes in the polymer of the selective dense layer in contact with ethylene glycol.

### 4.4 Mathematical Modeling of the Pervaporation Process

Modeling and simulation have become indispensable tools for engineers and researchers in the synthesis, analysis, and optimization of processes. Depending on the specific requirements, models of varying complexity can be employed, each exhibiting different levels of predictive accuracy and parameter determination demands.^41^

In this study, a global transmembrane model based on the solution–diffusion theory was employed. The model assumes thermodynamic equilibrium between the upstream liquid and the adjacent membrane surface, as well as between the downstream vapor and its corresponding membrane interface. Mass transport through the membrane is described by Fick’s law, with the fugacity difference of the permeant serving as the driving force. Two intrinsic properties characterize the membrane: the permeability (defined as the product of permeant flux and membrane thickness divided by the permeant driving force) and the permeance (defined as the permeant flux divided by the permeant driving force). The latter is typically used for asymmetric or composite membranes where the effective membrane thickness cannot be readily determined.^42^

The permeance of component *i* in the membrane, *Q*_*i*_, is defined with regard to the flux *J*_*i*_ as:

$$J_{i}=Q_{i}\left({\mathrm{f̂}}_{i}^{feed}-{\mathrm{f̂}}_{i}^{perm}\right)\approx Q_{i}\left(p_{i}^{o}x_{i}^{feed}\gamma_{i}-y_{i}p^{perm}\right)$$ 5

where *f̂*_*i*_^*feed*^ and *f̂*_*i*_^*perm*^ are the fugacities of component *i* in the feed mixture and in the permeate side of the membrane, respectively. The saturation vapor pressure (*p*_*i*_^*o*^) is obtained from the Antoine equation and the activity coefficients (γ_*i*_) have been obtained using the NRTL equation with parameters taken from the Aspen Plus V14 simulator database. The activities of the components in the liquid phase are calculated as:

$$a_{i}=x_{i}\gamma_{i}$$ 6

It is well established that, in systems where the polymer is swollen by permeant compounds, the partial permeation fluxes may exhibit a nonlinear dependence on the components’ activity. Consequently, the permeances are not constant but vary with the activity of the components.^43^ Accordingly, considering the various semiempirical models reported in the literature, the water permeance data were fitted to an equation that depends solely on the water activity in the feed mixture, with *A*_*i*_ and *B*_*i*_ being the fitting parameters of the equation:

$$Q_{water}=A_{1}\times \exp(B_{1}\times a_{water}):\;[Q_{i}]=\frac{\mathrm{mol}}{m^{2}\;h\;\mathrm{bar}}$$ 7

Regarding ethylene glycol, a similar approach was employed: its permeance can be expressed as a function of its activity in the feed mixture, as follows:

$$Q_{EG}=A_{2}\times \exp\left(B_{2}\times a_{EG}\right)$$ 8

In this study, the temperature dependence of membrane permeance in the proposed mathematical model was described through the Arrhenius-type equation (eq 9),

$$Q_{i,T}=Q_{i,\mathrm{To}}\exp\left(\frac{-E_{Q,i}}{RT}\right)$$ 9

It is also customary, for the case of pervaporation, to introduce the activation energy for the flux, *E*_*J,i*_ as follows:

$$J_{i,T}=J_{i,\mathrm{To}}\exp\left(\frac{-E_{J,i}}{RT}\right)$$ 10

which is obviously related to *E*_*Q,i*_ and to the feed properties in view of eq 5.

## Data Availability

The data underlying this study are available in the published article.
